# Supplementary material for: Patterns of Intron Gain and Loss in Fungi
Source: PLoS Biol. 2004 Nov 30;2(12):e422. doi: 10.1371/journal.pbio.0020422 (PMC532390; doi:10.1371/journal.pbio.0020422)
Supplement: Table S1 — Also available at http://genes.mit.edu/NielsenEtAl/. (4.3 MB ZIP). [file pbio.0020422.st001.zip › NielsenEtAl/html/149.html]

AN9461.1.NCU03796.1.MG07467.1.FG07381.1


```
 CLUSTAL W (1.82) Multiple Sequence Alignments - Introns Inserted


Sequence 1: NCU03796.1	430 aa
Sequence 2: FG07381.1	451 aa
Sequence 3: AN9461.1	442 aa
Sequence 4: MG07467.1	542 aa
Alignment Length: 587 aa
Number Identitical Residues: 202 aa
Alignment Score (without introns) 9836


MG07467.1 	MTRSSCSRVVSFAALGGRWS1QLWVVVRGRGTDSWNGRFSGDAAVKIV1PKRVAVPLNDV
NCU03796.1	--------------------~---------------------------~-----------
FG07381.1 	--------------------~---------------------------~-----------
AN9461.1  	--------------------~---------------------------~-----------
          	                                                            

MG07467.1 	QSSQSLPVARL2IL1EEKARGPQYLPKGEDYENTQTFNCGAIWDFDSLHGSSALEIDFSQ
NCU03796.1	-----------~--~---------------------------------------------
FG07381.1 	-------MPSI~AL~RQSRRCRLIDPRA-------YLTNTPRFSYTDIQSRAATNASWRP
AN9461.1  	-------MLRS~WP~NRCRRN-LRTQRG-------HLSVRVLHSESFVHN----PPPWRP
          	                           .         .     .     .          

MG07467.1 	EQLIPFYVPT2WVAKKARPVSLRQLMFFGRSLNEARLLSSANYVRTELPTR~LSHRIRDM
NCU03796.1	----------~--------------MVFGRSLTESRLISSANYVRTELPTR2IAHRIRDM
FG07381.1 	VSIL----DD2WVAKEARPISLRQLMVFGRSLTESRLISSANYVRTELPTR2IAHRIRDM
AN9461.1  	VSAL----DE2YVERQIRPISLRQLTFFGRTLTESRLLSSANYVRTELPTR2LAHRLRDI
          	 .             .  . :  .  .***:*.*:**:************* ::**:**:

MG07467.1 	QMLPYQVVSNQHIAEVYNMYWTAFDTFRKVKEVKSLADNDSFCEVISGMLKTHLTVIPKL
NCU03796.1	QRLPYVVVTNPHMNEVYDLYYSAFDTFRKVPEIKTLEDNDALCATIRSNLKAHLTVIPKL
FG07381.1 	QRLPYVVTTNNHIKEVYDLYYHAFDTFRKVKEVKTLDDNDKLCELISHNLKSHLTVIPQL
AN9461.1  	QKLPYAVVANPHFSLVYELYYKAFERFRTVPEIRTLDDNDRFCDILRKTLKEHLVVIPRL
          	* *** *.:* *:  **::*: **: **.* *:::* *** :*  :   ** **.***:*

MG07467.1 	AMGVLECSGLMDPQELNGFMNRILQS~RISRRVIAEQHVTLTQSFRNQTQPWSPPAGEAA
NCU03796.1	AMGILESAGLMDAKELDKFMNTILRS~RISRRVIAEQHLALTDTFH---APWFSP-----
FG07381.1 	AMGILECGGLMSPVDLDKFMNTILRS0RISRRVIAEQHLSLTETYN---SPNFSP-----
AN9461.1  	AMGVLECRGLLPAEAMDQFMNTLLRA0RISRRVIAEQHLALTETFN---SPWHFP-----
          	***:**. **: .  :: *** :*:: ***********::**:::.    *   *     

MG07467.1 	AVAATLGSASPIAESSDFVGNVLVRCVARDVVDRCAAAVHSLARSAHGEDVPLPEIRVVG
NCU03796.1	------GAKLS---ESEFIGEVFLKCVAKDVVERCGQAIRDIAQHAYGPDVAIPEIKIDG
FG07381.1 	------GAKLS---ESDFIGEVFIKCYAKDVVERCSKAITALARTTNGPDVQIPDITVDG
AN9461.1  	------GSQDRTDLNADFVGEVFLKCNAKEVVERCGKLVQDIIRQSSGSD-KIPEISVQG
          	      *:      .::*:*:*:::* *::**:**.  :  : : : * *  :*:* : *

MG07467.1 	HLSANFPFILSHLEYIIGELLRNSVDASVEQHQKSGKP-------------PPPIEVTIC
NCU03796.1	HLEANFPYILSHLEYIIGELLRNSVQAVVEKHQRYKEKVAAAGGKTDEIGPPPAIEVTIC
FG07381.1 	HLDASFPYILSHLEYIIGELLRNSVQAVIDRHQKTHPDPNNVK--------PPPVEITIC
AN9461.1  	HLDATFPYMLSHLEYIIGELLRNSIQAVSERYQGLNQT-------------PPPIEVLIC
          	**.*.**::***************::*  :::*                  **.:*: **

MG07467.1 	ESSQHVIIRVSDQGGGVARDMLPYLWSFSKGPHSDRLLQNLKHVPKMAATLQEVRAE---
NCU03796.1	ESQQHVIIRISDQGGGIQQDVLPYLWSFSKGPASQQLLTNLEQVPKMAATMQELKVEGST
FG07381.1 	ENQQHVIIRICDRGGGIPRAELPHLWSFSKGPQSQRRLNNLAQVPKMAATMQELHVE---
AN9461.1  	EAPQHVIMRVSDQGGGIPREILPYLWSFTKGPHSKVRLENLGQVPAMAATLQELSVS---
          	*  ****:*:.*:***: :  **:****:*** *.  * ** :** ****:**: ..   

MG07467.1 	--------------------DDGILSIPQITT---------DQSVLSSLSSLSSRPPNLK
NCU03796.1	SSEAGTTVGKLQQQRQEQQQQQQQQQKQQIKGIHNGNNFPGDPGYCNSLATLTSRPPNLR
FG07381.1 	----------------------EELGRADLK----------VPPYQSSLSSLTSRPPNLR
AN9461.1  	-------------------SDIKHADKETFR--------------ESSLDTLTSRPPDLR
          	                   ..        :                .** :*:****:*:

MG07467.1 	LGVGLPLSRVYAEYWAGSLELHSLEGYGVDTFLQISKLGNKNEQLATRASMDAV
NCU03796.1	LGMGLPLSRVYAEYWAGSLALHSLEGYGVDAFLQISKLGNKNEQLTTRATMDAV
FG07381.1 	LGMGLPLSRVYAEYWAGNLDLHSLEGYGTDMFLQISRLGNKNEQLTTRASMDAL
AN9461.1  	LGIGLPMSRVYAEYWAGSLELHSLEGYGVDAFLQISKLGNKNEQVTTRAAIDAV
          	**:***:**********.* ********.* *****:*******::***::**:
```
